# Recent Advances in Natural Products Derived from Marine Echinoderms and Endophytic Microbes: Chemical Insights and Therapeutic Potential

**DOI:** 10.3390/md23010033

**Published:** 2025-01-10

**Authors:** Shuangyu Li, Yan Xiao, Qiang Li, Mingzhi Su, Yuewei Guo, Xin Jin

**Affiliations:** 1 Shandong Laboratory of Yantai Drug Discovery, Bohai Rim Advanced Research Institute for Drug Discovery, Yantai 264117, China; shuangyu0404@163.com (S.L.); lq1030561007@163.com (Q.L.); mzsu@baridd.ac.cn (M.S.); 2 School of Pharmaceutical Engineering, Shenyang Pharmaceutical University, Shenyang 124221, China; xiaoyan@syphu.edu.cn; 3 School of Medicine, Shanghai University, Shanghai 200444, China

**Keywords:** echinoderms, marine natural product, triterpene glycoside, steroid glycosides

## Abstract

Echinoderms, a diverse group of marine invertebrates including starfish, sea urchins, and sea cucumbers, have been recognized as prolific sources of structurally diverse natural products. In the past five years, remarkable progress has been made in the isolation, structural elucidation, and pharmacological assessment of these bioactive compounds. These metabolites, including polysaccharides, triterpenoids, steroids, and peptides, demonstrate potent bioactivities such as anticancer, anti-inflammatory, antiviral, and antimicrobial effects, providing valuable insights and scaffolds for drug discovery. This review highlights the structural diversity and biological activities of natural products derived from echinoderms over the last five years, with a particular focus on their structure–activity relationships and therapeutic potential. It also outlines the prospects and challenges for future research, aiming to stimulate further exploration in marine drug discovery.

## 1. Introduction

Echinoderms, classified within the phylum Echinodermata, constitute a diverse group of exclusively marine invertebrates, including sea cucumbers (Holothuroidea), sea stars (Asteroidea), brittle stars (Ophiuroidea), sea urchins (Echinoidea), and feather stars (Crinoidea). These organisms are distributed widely across global oceanic environments, from shallow coastal waters to the deep sea trenches [1]. Beyond their ecological importance, echinoderms have attracted increasing interest due to their rich chemical compositions and the bioactive compounds they produce, which hold potential for pharmaceutical and biomedical applications [2,3].

The chemical constituents of echinoderms are remarkably diverse, encompassing a variety of secondary metabolites such as saponins, peptides, polysaccharides, steroids, fatty acids, and carotenoids [4,5,6,7,8]. Notably, the saponins found in sea cucumbers and sea stars, specifically triterpene glycosides and steroidal glycosides, have been extensively studied for their potent biological activities. These compounds exhibit a broad range of pharmacological effects, including antitumor, anticoagulant, antiviral, immunomodulatory, and anti-inflammatory activities [9].

Sea cucumbers are particularly rich in triterpene glycosides, known as holothurins and holotoxins [10], which have demonstrated significant cytotoxicity against various cancer cell lines. Research indicates that these glycosides can induce apoptosis and inhibit metastasis, highlighting their potential as anticancer agents [11]. Additionally, sulfated polysaccharides derived from sea cucumber, such as fucosylated chondroitin sulfates, possess anticoagulant and antithrombotic properties, making them promising candidates for the development of new anticoagulant drugs [12].

Sea stars produce a range of steroidal compounds, including asterosaponins and polar steroids, which have exhibited antiviral, antibacterial, and antifungal activities. These compounds have also shown cytotoxic effects against tumor cells, further emphasizing the pharmaceutical potential of echinoderm-derived metabolites. The unique structures of these molecules often differ from those found in terrestrial organisms, offering novel scaffolds for drug development [13,14,15].

The exploration of echinoderm chemical composition not only contributes to the discovery of new bioactive substances, but also enhances our understanding of ecological roles in marine environments. Many of these metabolites serve as chemical defenses against predators, parasites, and microbial infections, highlighting the evolutionary significance of these chemical adaptations [16].

Given the increasing drug resistance and the ongoing demand for new therapeutic agents, echinoderms represent a valuable source of novel compounds with potential applications in medicine. Advances in analytical techniques and marine biotechnology have facilitated the isolation and characterization of these complex molecules, paving the way for drug discovery and development [17].

This review provides a comprehensive overview of the chemical constituents of echinoderms and their associated biological activities. By synthesizing current research findings, we aim to highlight the pharmacological potential of echinoderm-derived compounds and encourage further exploration in this promising field.

## 2. The Structure and Activity of Compounds Isolated from Echinoderms

### 2.1. Alkaloids

**Figure 1:**
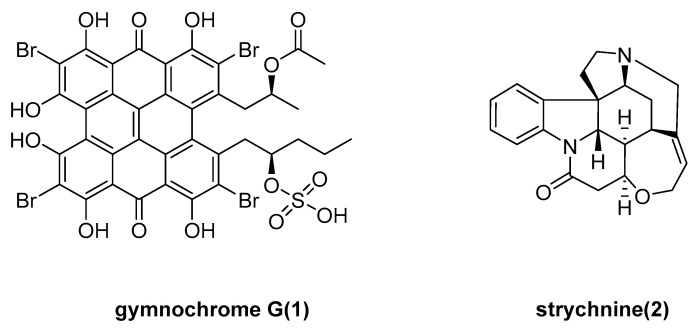
The structure of gymnochrome G (**1**) and strychnine (**2**).

Niels Karschin et al. determined the structure of the new alkaloid gymnochrome G (**1**) and strychnine (**2**) (Figure 1) isolated from the deep sea crinoid *Hypalocrinus naresianus*, from a sample collected from Japan [18,19]. However, the activities of the compounds have not been discovered.

**Figure 2:**
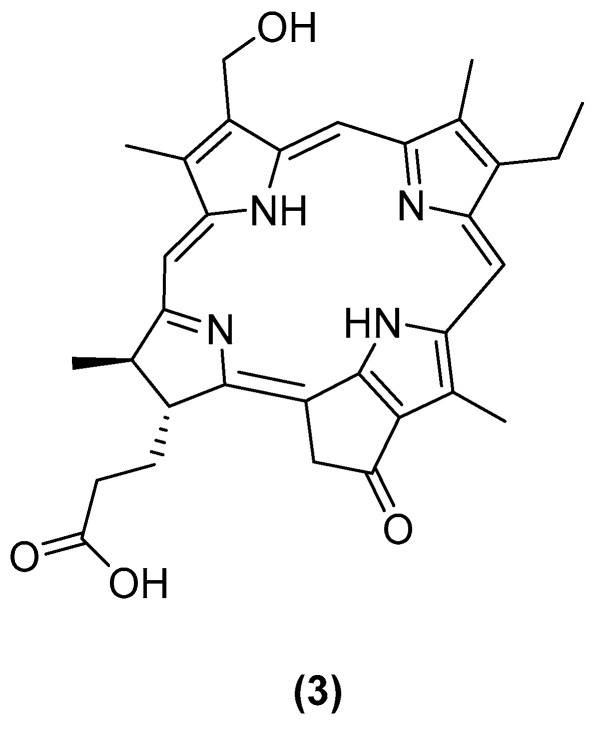
The structure of compound **3**.

Klimenko et al. reported an unusual porphyrin derivative (**3**, Figure 2), isolated in a single step from the North Pacific brittle star *Ophiura sarsii*, which exhibited potent cytotoxic activity against triple-negative breast cancer (TNBC), with an IC_50_ of approximately 30 µM. This finding represents the first report of a porphyrin from an Ophiuroidea species. Given that porphyrins are well-known as photosensitizers in photodynamic therapy, this discovery may pave the way for a novel, natural-based approach to targeted anticancer treatments [20].

**Figure 3:**
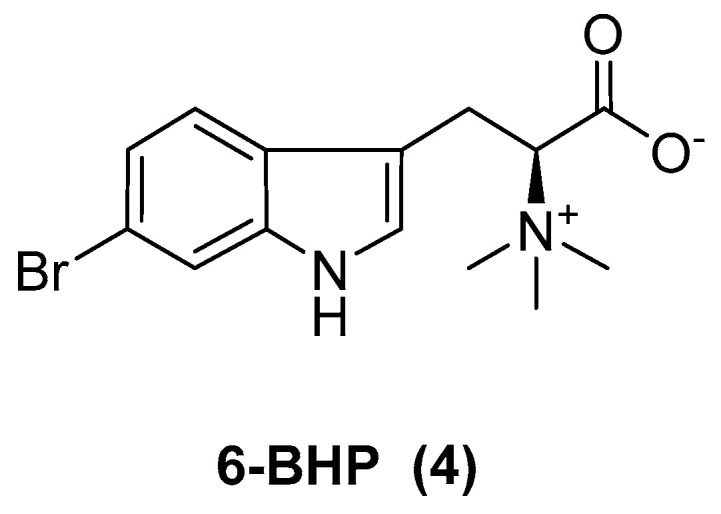
The structure of 6-BHP.

L-6-bromohypaphorine (6-BHP, Figure 3) was isolated from the sponge *Pachymatisma johnstonii* for the first time [21]. Dai et al. were the first to isolate 6-BHP from the sea cucumber *Apostichopus japonicus*, and evaluated its anticancer properties. The 6-BHP exhibited significant anticancer activity, particularly against the A549 tumor-cell lines, with an IC_50_ value of 197.1 ± 7.4 µM. Additionally, 6-BHP downregulated B-cell lymphoma-extra large (Bcl-xL) while upregulating both Bcl-2-associated X protein (Bax) and caspase-3 in a dose- and time-dependent manner. It induced apoptosis via the mitochondrial-mediated pathway [22].

**Figure 4:**
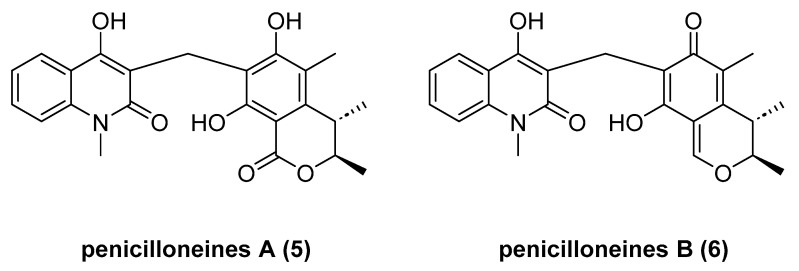
The structure of penicilloneines A (**5**) and B (**6**).

Penicilloneines A (**5**) and B (**6**) (Figure 4), quinolone−citrinin hybrids, were isolated from the starfish-derived fungus *Penicillium* sp. GGF16-1-2 [23]. Penicilloneines A and B share a common 4-hydroxy-1-methyl-2(1H)-quinolone unit; however, they differ in terms of citrinin moieties. Penicilloneines A and B exhibited antifungal activities against *Colletotrichum gloeosporioides*, with LC_50_ values of 0.02 and 1.51 μg/mL, respectively. Penicilloneines A could inhibit cell growth and promote cell vacuolization and consequent disruption of the fungal cell walls via the upregulation of nutrient-related hydrolase genes and the downregulation of their synthase genes.

**Figure 5:**
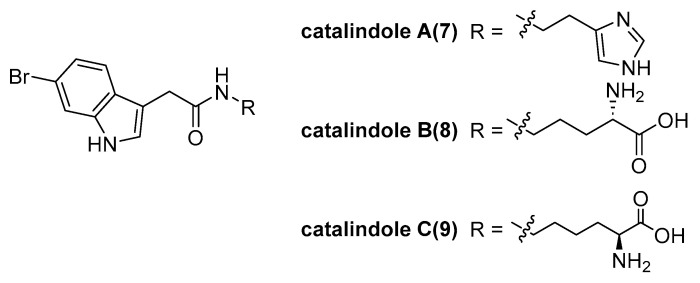
The structure of catalindoles A–C (**7**–**9**).

Ryuga Matsuta et al. isolated new brominated indole alkaloids, catalindoles A–C (**7**–**9**, Figure 5), from the arms of *Thromidia catalai* [12], which revealed the predator–prey relationship between *T. catalai* and *Theonella* sponges, based on their chemical components. It was noted that *T. catalai* feeds not only on *T. swinhoei* (chemotype Y) but also on *T.* swinhoei (chemotype W).

Although reports on alkaloid compounds in echinoderms are relatively limited, some novel structures, such as brominated indoles and rare porphyrin derivatives, have demonstrated unique physiological activities and potential medicinal value. Future research could focus on exploring their mechanisms of action, such as their effects on apoptosis or cellular signaling pathways, and conducting more in vivo evaluations to determine their pharmacological safety profiles.

### 2.2. Triterpene and Triterpene Glycosides

Triterpenes and their glycosides are the most extensively studied secondary metabolites in echinoderms such as sea cucumbers. The current research indicates that they exhibit significant activities in areas such as antitumor, immunomodulatory, and anti-inflammatory effects. Most triterpene glycosides exert their biological functions by interfering with multiple signaling pathways, such as NF-κB, PI3K/Akt, and MAPK [24,25,26]. For instance, certain triterpene glycosides can bind to key receptor proteins, thereby inhibiting pro-inflammatory cytokines or inducing tumor-cell apoptosis. In addition, recent reports have indicated that tri-iron glycoside P57 can rapidly induce hypothermia in mice while significantly reducing energy expenditure [27].

**Figure 6:**
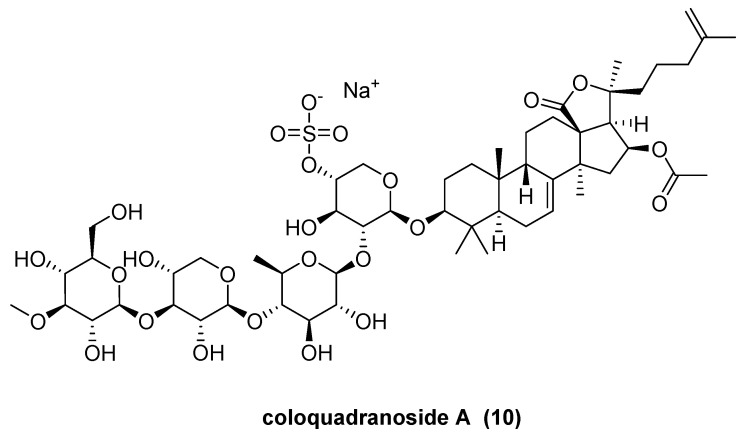
The structure of coloquadranoside A (**10**).

Yang, W.-S. et al. reported a new sulfated triterpene glycoside, coloquadranoside A (**10**, Figure 6), featuring a holostane-type triterpene aglycone, and which is isolated from the sea cucumber *Colochirus quadrangularis* [28]. Four known triterpene glycosides—philinopside A, B, and E, and pentactaside B—were also obtained; these compounds were first isolated from the sea cucumber *Pentacta quadrangularis* [29,30,31]. Coloquadranoside A inhibited certain fungi in vitro and exhibited considerable cytotoxicity (IC_50_ of 0.46–2.03 μM) against eight human tumor-cell lines. Additionally, coloquadranoside A suppressed the migration and proliferation of human colon cancer cell line HCT-116 and human microvascular endothelial cell line HMEC-1 cells while accelerating their apoptosis by inhibiting tumor neovascularization through the tyrosine kinase autophosphorylation pathway. In vivo, administration of coloquadranoside A at doses of 50–500 mg/kg ameliorated the immune organ index, thereby enhancing humoral and adaptive immune functions under immunosuppressed conditions.

**Figure 7:**
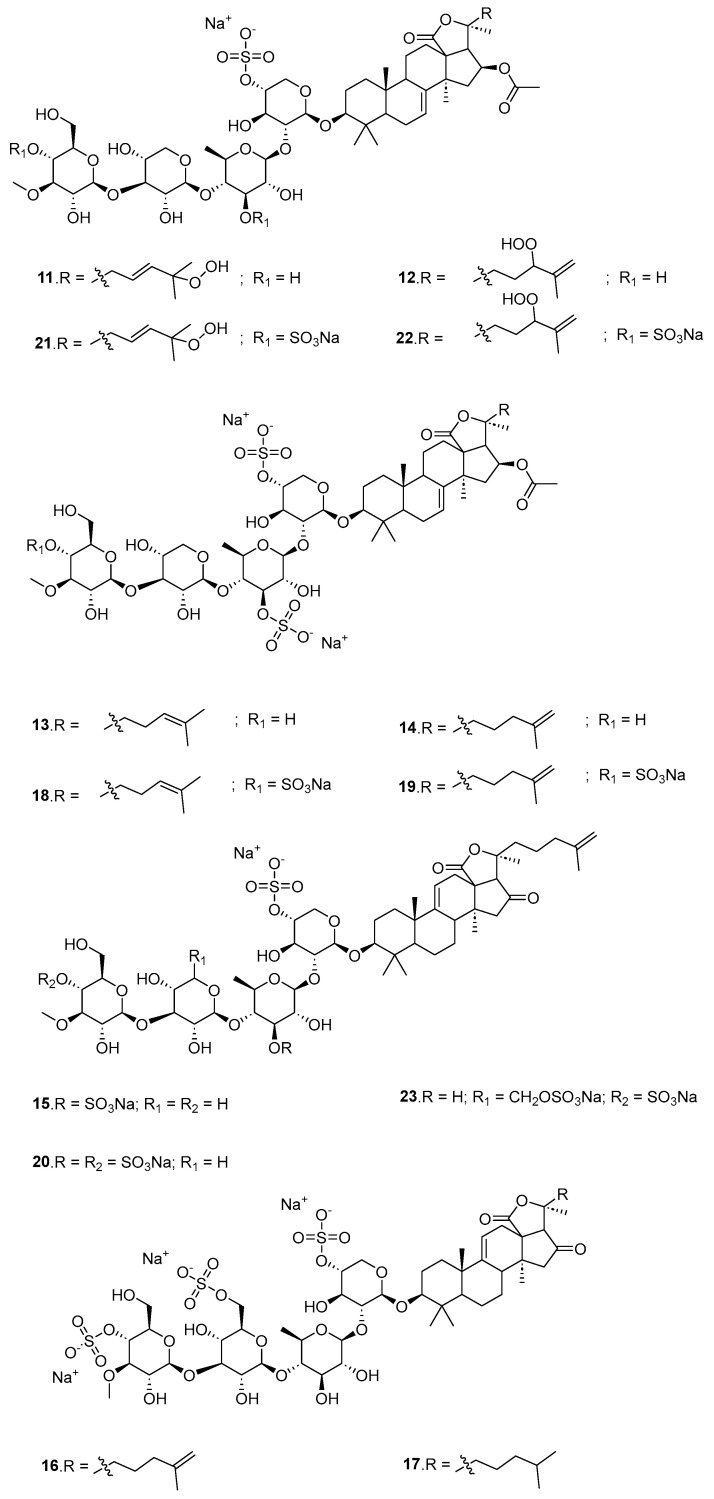
The structure of quadrangularisoside A (**11**), quadrangularisoside A_1_ (**12**), quadrangularisoside B (**13**), quadrangularisoside B1 (**14**), quadrangularisoside B2 (**15**), quadrangularisoside C (**16**), quadrangularisoside C1 (**17**), quadrangularisoside D (**18**), quadrangularisoside D1 (**19**), quadrangularisoside D2 (**20**), quadrangularisoside D3 (**21**), quadrangularisoside D4 (**22**), and quadrangularisoside E (**23**).

Thirteen new mono-, di-, and trisulfated triterpene glycosides, quadrangularisosides A–E (**11**–**23**, Figure 7) have been isolated from the sea cucumber *Colochirus quadrangularis*, which was collected in Vietnamese waters [32]. All of the compounds were rather strong hemolytics (ED_50_ around 0.1 to 5 μM). The structural features that most affect the bioactivity of the glycosides are the presence of hydroperoxy groups in the side chains and the quantity of sulfate groups. Quadrangularisosides A_1_ (**12**), C (**16**), C_1_ (**17**), and E (**23**) possessed strong inhibitory activity on colony formation in HT-29 cells. Due to the synergic effects of these glycosides (0.02 µM) and radioactive irradiation, a decreasing number of colonies was detected. Glycosides **11**, **13**, and **19** enhanced the effect of radiation by about 30%.

**Figure 8:**
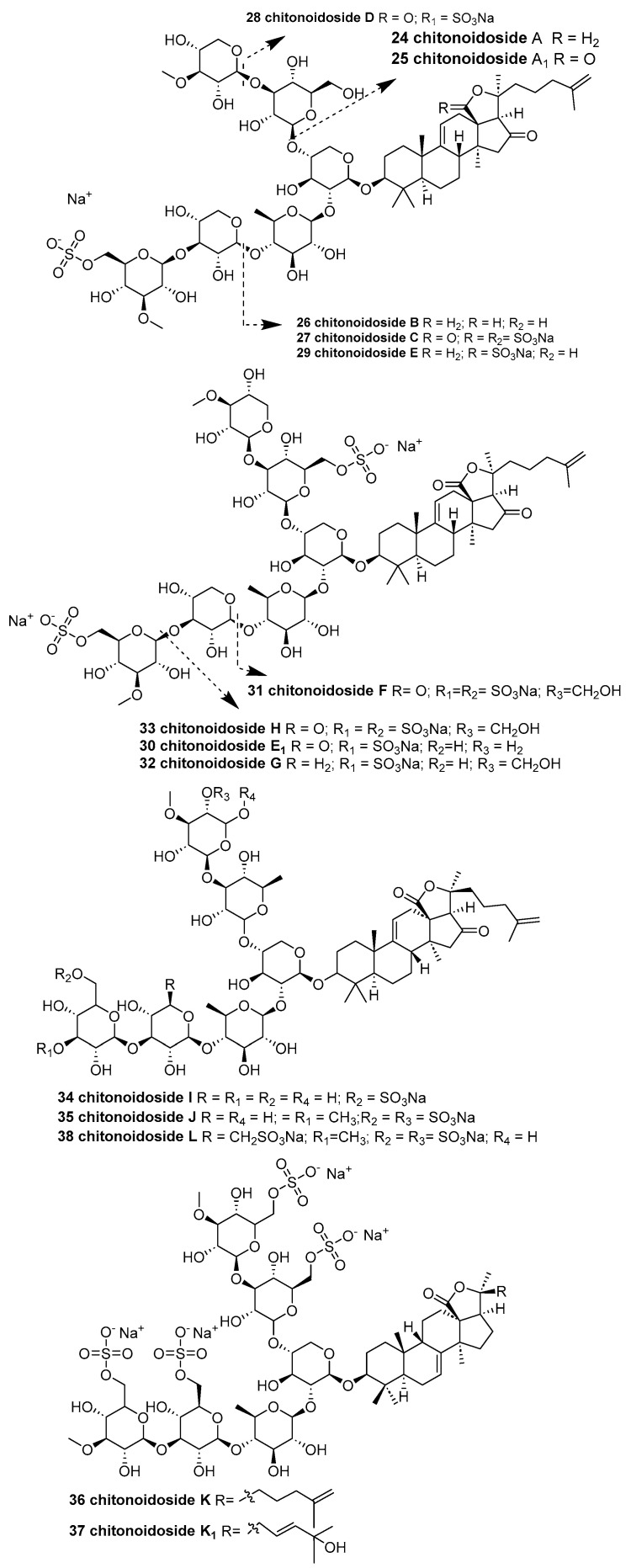
The structure of chitonoidosides A (**24**), A_1_ (**25**), B (**26**), C (**27**), D (**28**), E (**29**), E_1_ (**30**), F (**31**), G (**32**), H (**33**), I (**34**), J (**35**), K (**36**), K_1_ (**37**), and L (**38**).

Silchenko et al. reported six new sulfated triterpene glycosides—chitonoidosides A (**24**), A_1_ (**25**), B (**26**), C (**27**), D (**28**), and E (**29**) (Figure 8)—isolated from the Far Eastern sea cucumber *Psolus chitonoides*, collected near Bering Island (Commander Islands) at a depth of 100–150 m [33]. These glycosides, which contain one or two sulfate groups, exhibit structural diversity in their aglycones (featuring 7(8)- or 9(11)-double bonds) and carbohydrate moieties, differing in both sugar residue composition and the positions of the sulfate groups. Notably, compounds bearing four sulfate groups (e.g., chitonoidosides B (**26**) and D (**28**)) demonstrated significant hemolytic and cytotoxic activities against various cancer cell lines. Among them, chitonoidoside D (**28**) displayed the most pronounced cytotoxic effect, with an EC_50_ value of 1.86 ± 0.23 μM against HL-60 cells (human promyelocytic leukemia cell line). This potency was attributed to the presence of a pentasaccharide disulfated sugar chain in combination with a holostane aglycone. Surprisingly, chitonoidosides A (**24**) and B (**26**), which incorporate a novel aglycone lacking a γ-lactone ring, exhibited cytotoxicities comparable to those of known holostane-type glycosides. In contrast, chitonoidoside C (**27**) was less cytotoxic, likely due to its distinct carbohydrate chain architecture and the presence of a sulfate group at C-4 in 3-O-MeXyl4.

Four new triterpene disulfated glycosides, chitonoidosides E_1_ (**30**), F (**31**), G (**32**), and H (**33**) (Figure 8), were discovered in samples of the Far Eastern sea cucumber *Psolus chitonoides* collected near Bering Island (Commander Islands) at depths of 100–150 m [34]. These compounds include two hexaosides differing in their terminal (sixth) sugar residue, one pentaoside, and one tetraoside. They are notable for their oligosaccharide chains with shortened bottom semi-chains, a rare feature among sea cucumber glycosides. The hemolytic activities of compounds **28**–**31** and chitonoidoside E (**29**) were investigated against human erythrocytes. In addition, their cytotoxic effects were tested against several human cancer cell lines, including HeLa (a human cervical adenocarcinoma cell line), DLD-1 (a human colorectal adenocarcinoma cell line), and THP-1 (a human monocytic leukemia cell line). The glycosides with hexasaccharide chains—E_1_ (**30**), G (**32**), and chitonoidoside E—exhibited the highest hemolytic activities (ED_50_ = 1 μM) against erythrocytes. A similar trend was observed in their cytotoxicity towards HeLa cells, albeit with moderate effects. Both THP-1 cells and erythrocytes showed comparable sensitivity to these glycosides; however, the activities of chitonoidosides E (**28**) and E_1_ (**30**) differed significantly from that of G (**32**) against THP-1 cells. In contrast, the chitonoidoside F (**31**), characterized by its shortened bottom semi-chain, displayed the weakest membranolytic effect in the series.

Five new triterpene di-, tri-, and tetrasulfated hexaosides, designated chitonoidosides I (**34**), J (**35**), K (**36**), K_1_ (**37**), and L (**38**) (Figure 8), were isolated from the Far Eastern sea cucumber *Psolus chitonoides*, collected near Bering Island (Commander Islands) at depths of 100–150 m [35]. Compounds (**34**–**38**) were evaluated for their hemolytic activities and cytotoxicities against human cancer cell lines. Among them, the hexaosides chitonoidoside K (**36**), with an EC_50_ value of 6.77 ± 0.44 μM, and chitonoidoside L (**38**), with an EC_50_ value of 7.89 ± 0.44 μM—each containing four sulfate groups—were the most active against tumor cells in all tests.

**Figure 9:**
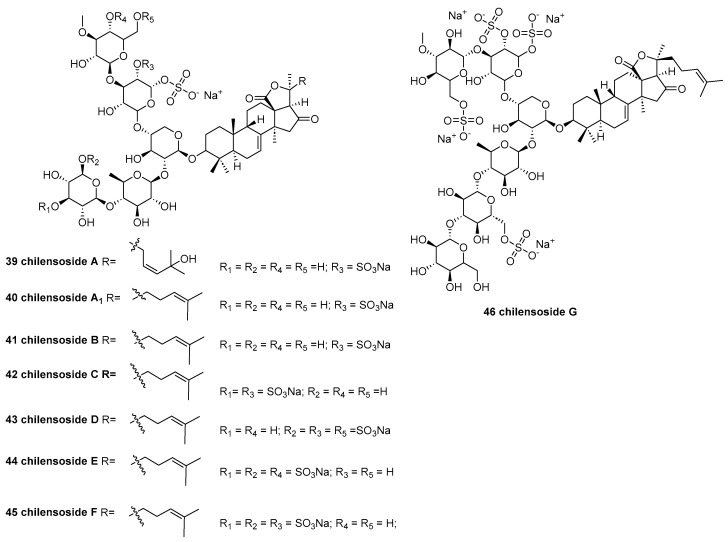
The structure of chilensosides A (**39**), A_1_ (**40**), B (**41**), C (**42**), D (**43**), E (**44**), F (**45**), and G (**46**).

Eight new triterpene chilensosides, A (**39**), A_1_ (**40**), B (**41**), C (**42**), and D (**43**) [36]; and E (**44**), F (**45**), and G (**46**) (Figure 9) [37], were isolated from the Far Eastern sea cucumber *Paracaudina chilensis*. The cytotoxic activities of the compounds against human cancer cell lines SH-SY5Y, HeLa, DLD-1, HL-60, and THP-1 were tested. Compounds (**40**–**42**) demonstrated significant cytotoxic activity against these cell lines, while chilensoside B (**41**) exhibited a remarkably low EC_50_ of 5.68 ± 0.05 μM against HL-60 cells.

**Figure 10:**
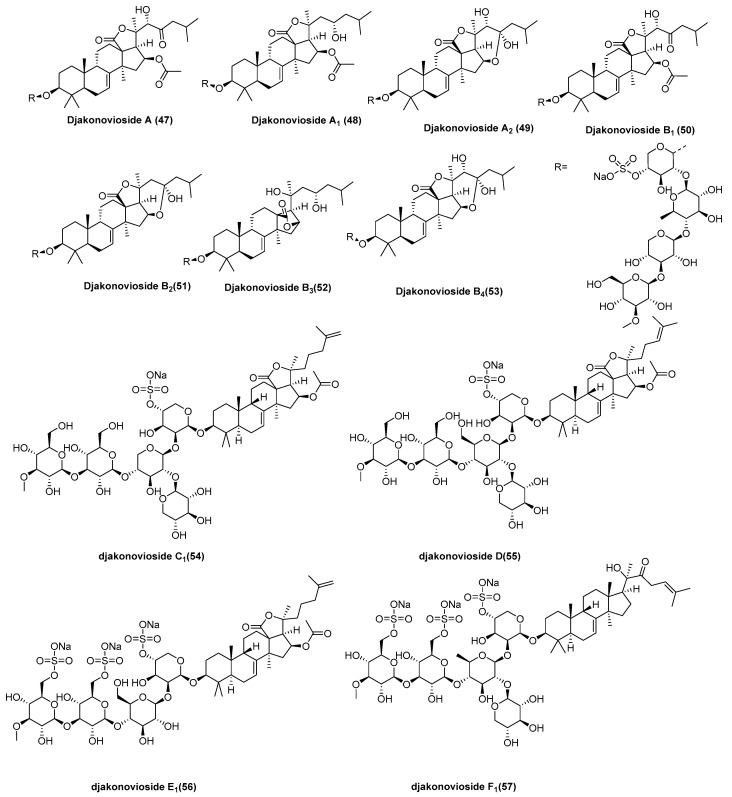
The structure of triterpene glycosides: djakonoviosides A (**47**), A_1_ (**48**), A_2_ (**49**), B_1_ (**50**), B_2_ (**51**), B_3_ (**52**), B_4_ (**53**), C_1_ (**54**), D_1_ (**55**), E_1_ (**56**), and F_1_ (**57**).

Eleven new monosulfated triterpene glycosides, djakonoviosides A (**47**), A_1_ (**48**), A_2_ (**49**), and B_1_–B_4_ (**50**–**53**) [38]; and C_1_ (**54**), D_1_ (**55**), E_1_ (**56**), and F_1_ (**57**) [39], as well as three known glycosides, okhotoside A1-1, cucumarioside A0-1 (Figure 10), and Frondoside D were isolated from the Far Eastern sea cucumber *Cucumaria djakonovi* (*Cucumariidae, Dendrochirotida*). Okhotoside A1-1 represents the first isolation from *C. okhotensis* [40]. Cucumarioside A0-1 was isolated from *C. japonica* [41], and frondoside D was initially isolated from *C. frondose* [42].The hemolytic and antitumor activities of the compounds were investigated using erythrocytes and various cancer cell lines, including breast cancer cells (MCF-7, T-47D, and triple-negative MDA-MB-231) and the HL-60 and HEK-293 cell lines. Djakonovioside E_1_ (**56**) demonstrated selective cytotoxicity against ER-positive MCF-7 cell lines, with an EC_50_ value of 1.52 ± 0.14 μM, and the triple-negative MDA-MB-231 cell lines, with an EC_50_ value of 2.19 ± 0.17 μM, while exhibiting no toxic effect on normal mammary epithelial cells (MCF-10A).

**Figure 11:**
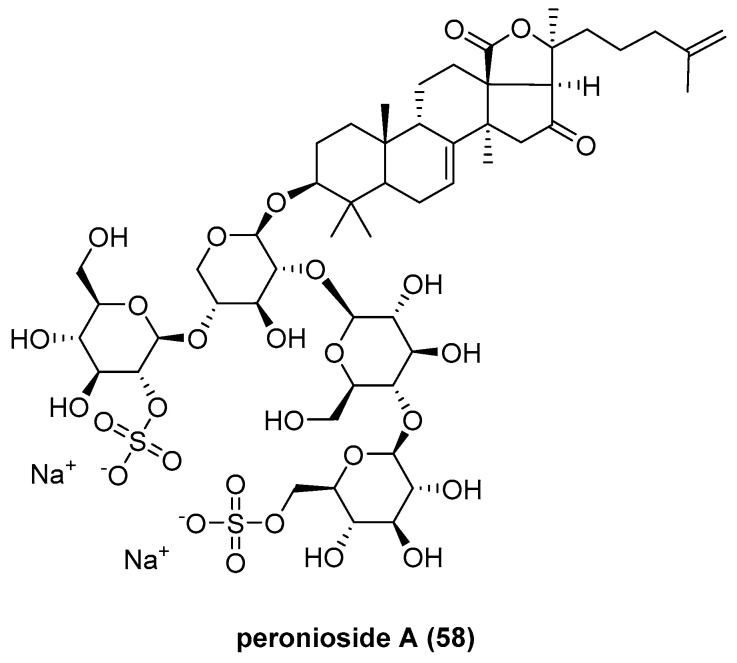
The structure of peronioside A (**58**).

Eight sulfated triterpene glycosides, peronioside A (**58**) [43] and psolusosides A, B, G, I [44], L, N, and P (Figure 11) [45], were isolated from the sea cucumber *Psolus peronii*. This discovery underscores the phylogenetic and systematic proximity of these echinoderm species. Among these glycosides, peronioside A has been characterized as a novel compound; however, it has not demonstrated significant bioactivity in the assays conducted.

**Figure 12:**
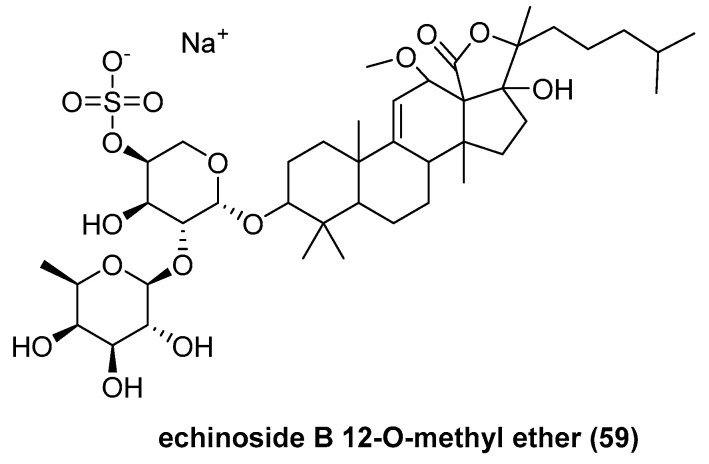
The structure of triterpene glycoside echinoside B 12-O-methyl ether (**59**).

Four sulfated holostan-type triterpene glycosides were isolated by Hawas, U. W. et al. from sea cucumber *Holothuria atra* collected from southern Corniche of Jeddah, Saudi Arabia [46], including a new echinoside B 12-O-methyl ether (**59**) (Figure 12) and three known compounds, echinoside B, 24-dehydroechinoside B [47], and holothurin B [48]. Compound **59** at 25 μg/mL displayed weak antioxidant DPPH radical scavenging activities, of 21%, and moderate antitumor activity for Ehrlich ascites carcinoma (EAC) cells, with 29.95%

**Figure 13:**
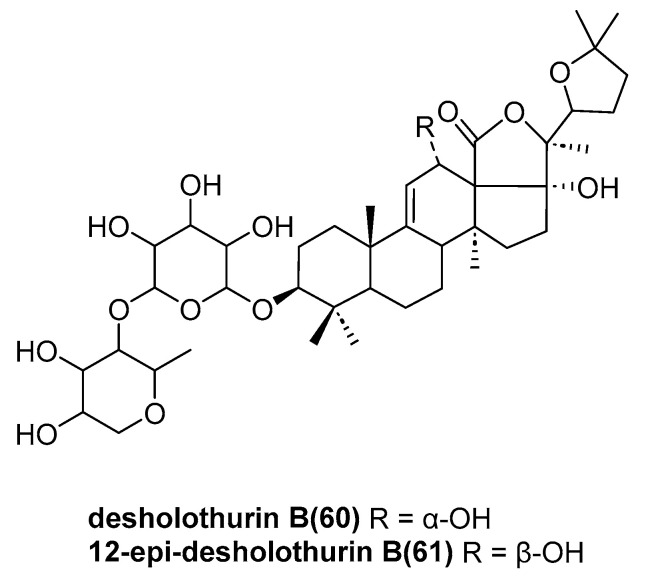
The structure of triterpene glycosides: desholothurin B (**60**) and 12-epi-desholothurin B (**61**).

Puspitasari, Y. E. and coworkers identified two previously unreported compounds, including the triterpene glycosides desholothurin B (**60**) and the novel 12-epi-desholothurin B (**61**) (Figure 13), from the sea cucumber *Holothuria atra* [49]. The activities of these compounds were not been disclosed.

**Figure 14:**
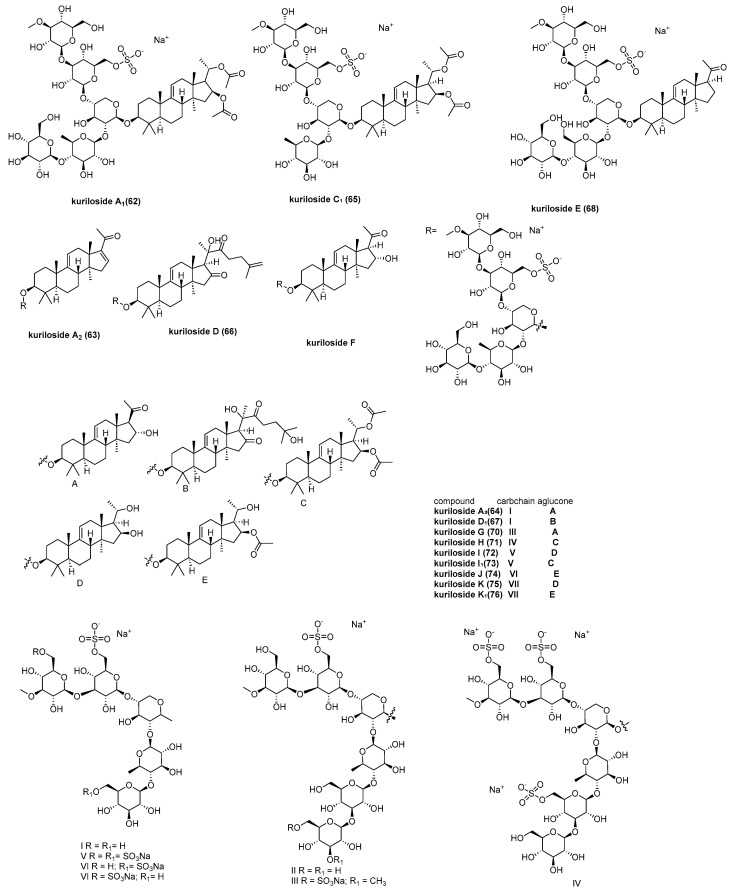
The structure of kurilosides A_1_ (**62**), A_2_ (**63**), A_3_ (**64**), C_1_ (**65**), D (**66**), D_1_ (**67**), E (**68**), F (**69**), G (**70**), H (**71**), I (**72**), I_1_ (**73**), J (**74**), K (**75**), and K_1_ (**76**).

Fifteen new monosulfated triterpene glycosides, kurilosides A_1_ (**62**), A_2_ (**63**) [50], A_3_ (**64**), C_1_ (**65**), D (**66**), D_1_ (**67**), E (**68**), F (**69**), G (**70**), H (**71**), I (**72**), I_1_ (**73**), J (**74**), K (**75**), and K_1_ (**76**) (Figure 14) [51], were isolated from the sea cucumber *Thyonidium* (*Duasmodactyla*) *kurilensis* (*Levin*) (*Cucumariidae, Dendrochirotida*) by Silchenko, A. S. et al. The cytotoxic activities of compounds (**62**–**76**) were evaluated against mouse neuroblastoma Neuro 2a cells, normal epithelial JB-6 cells, and erythrocytes. Among them, the trisulfated hexaoside kuriloside H (**71**) exhibited the highest cytotoxicity to JB-6, with an EC_50_ of 4.63 ± 0.08 μM.

**Figure 15:**
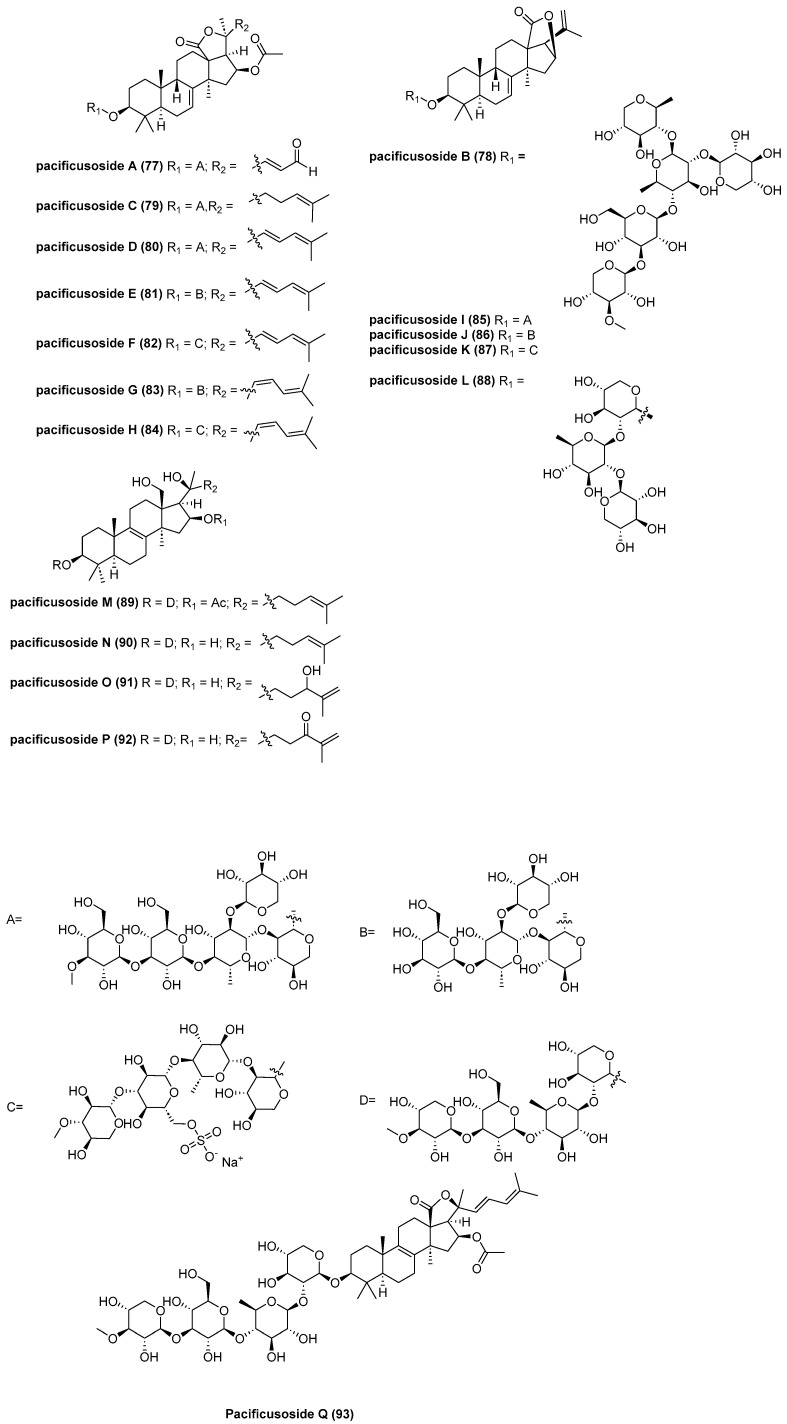
The structure of pacificusosides A–Q: A (**77**), B (**78**), C (**79**), D (**80**), E (**81**), F (**82**), G (**83**), H (**84**), I (**85**), J (**86**), K (**87**), L (**88**), M (**89**), N (**90**), O (**91**), P (**92**), and Q (**93**).

Timofey, V. et al reported 17 new compounds, pacificusosides A–C (**77**–**79**) [52], pacificusosides D–K (**80**–**87**) [53], and pacificusosides L–Q (**88**–**93**) [54] (Figure 15). The cytotoxic activities of **80**–**87** against non-cancerous mouse epidermal cells JB6 Cl41, SK-MEL-2, SK-MEL-28, and RPMI-7951 were determined by MTS assay. Compounds **80**, **82**, **84**, and **87** showed potent cytotoxicity against these cell lines, with IC_50_ values around 6 μM.

**Figure 16:**
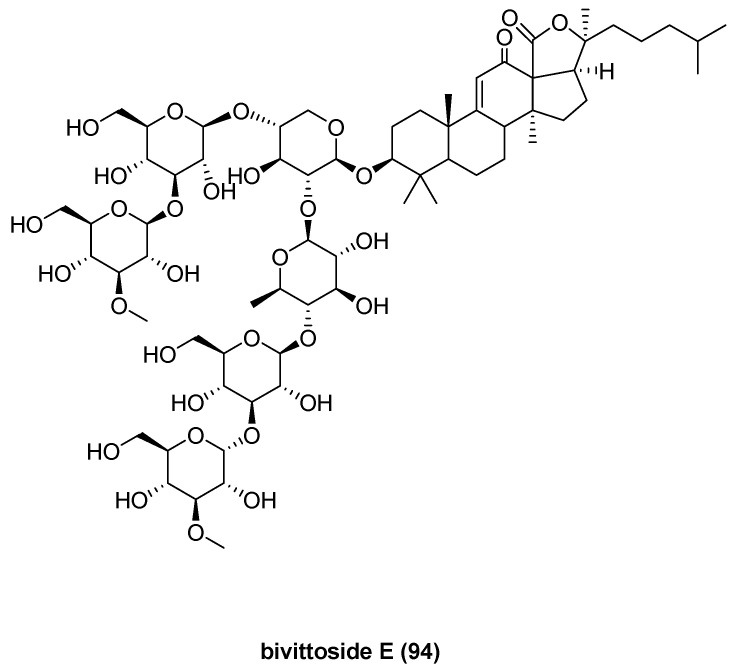
The structure of bivittoside E (**94**).

Choi, H. et al. identified antagonists of the transient receptor potential ankyrin 1 (TRPA1) channel. Screening 393 marine invertebrate extracts revealed that the edible sea cucumber *Bohadschia vitiensis* exhibited significant TRPA1 antagonistic activity. Bioassay-guided fractionation led to the isolation of six triterpene glycosides, including a novel compound named bivittoside E (**94**) and five known compounds, impatienside A [55]; arguside C [56]; bivittoside C, D [57,58]; and holothurinoside H [59] (Figure 16). Compound **94** demonstrated potent inhibitory activity against TRPA1, with an IC_50_ of 2.26 ± 0.04 μM [60].

**Figure 17:**
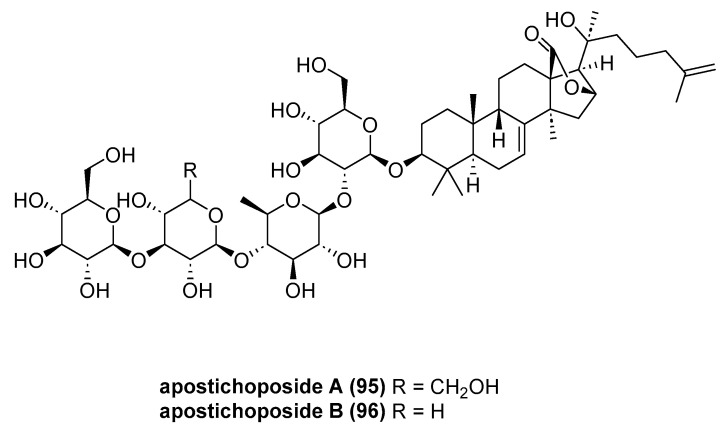
The structure of apostichoposide A (**95**) and apostichoposide B (**96**).

Hang, X.-M. et al. report the isolation of two novel glycosides, apostichoposide A (**95**) and B (**96**) (Figure 17), from the viscera of *Apostichopus japonicus* sea cucumbers collected in the Bohai Sea. Cytotoxic activities of the two compounds were evaluated against three human cancer cell lines. It was demonstrated that apostichoposide A displayed adequate cytotoxic activity against MGC-803 and PC-3M cell lines, with IC_50_ values of 57.22 ± 3.72 and 76.71 ± 4.79 μg/mL, respectively [61].

**Figure 18:**
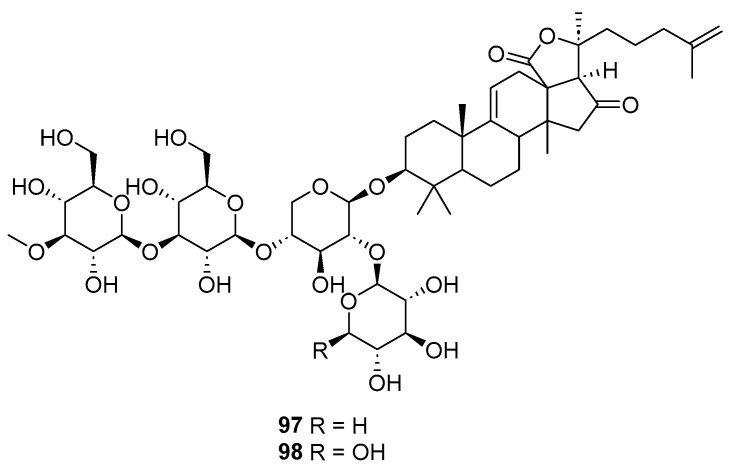
The structures of compounds **97** and **98**.

Two undescribed triterpene glycosides (**97** and **98**, Figure 18) [62], together with a known cladoloside A [63], were isolated from sea cucumbers (*Apostichopus japonicus*) in the Yellow Sea of China. Compounds **97** and **98** exhibited embryotoxic effects, as evidenced by their respective 97 h post-fertilization lethal concentrations (97 hpf-LC50) of 0.289 μM and 0.536 μM. These values indicate a significant impact on embryonic development at these concentrations.

### 2.3. Steroid and Steroid Glycosides

Steroids and their glycosides also hold promise in anti-inflammatory, antitumor, and immunoregulatory applications. Particularly, steroids with multiple hydroxyl or sulfate groups often exhibit strong cytotoxic or membrane-disrupting activities. Like triterpene glycosides, common molecular mechanisms of steroid compounds include regulating the cell cycle, inducing apoptosis, and affecting membrane permeability [9]. Enhancing research on the isolation, identification, and mechanisms of action of steroid glycosides will contribute to expanding their clinical application potential.

**Figure 19:**
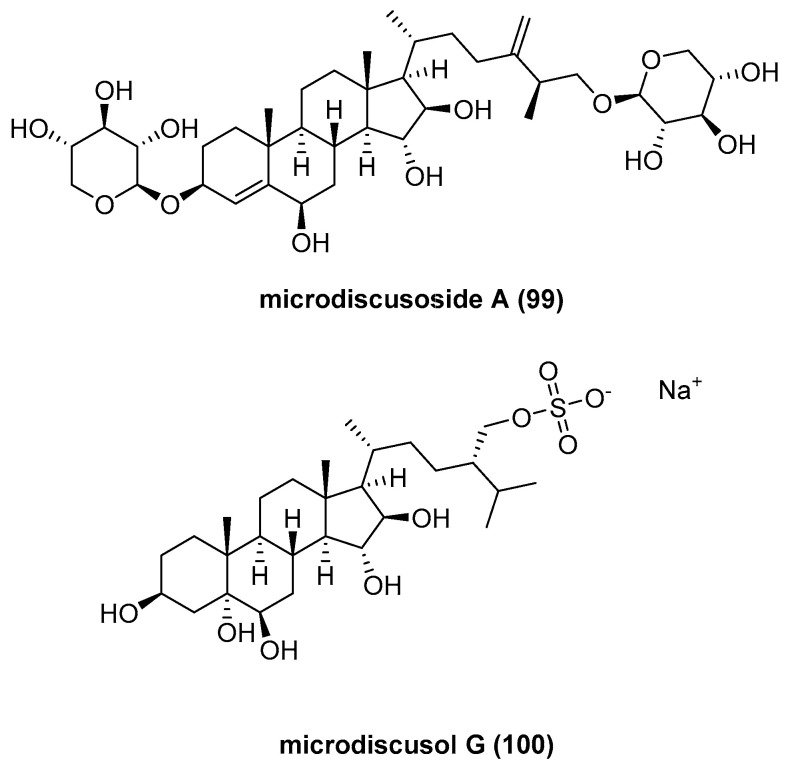
The structure of microdiscusoside A (**99**) and microdiscusol G (**100**).

Two new natural compounds, sulfated polyhydroxysteroid, microdiscusoside A (**99**), and polyhydroxysteroid bioside, microdiscusol G (**100**) (Figure 19), were isolated from the Arctic starfish *Asterias microdiscus* [64]. Owing to insufficient yields of both compounds, their activities were not tested.

**Figure 20:**
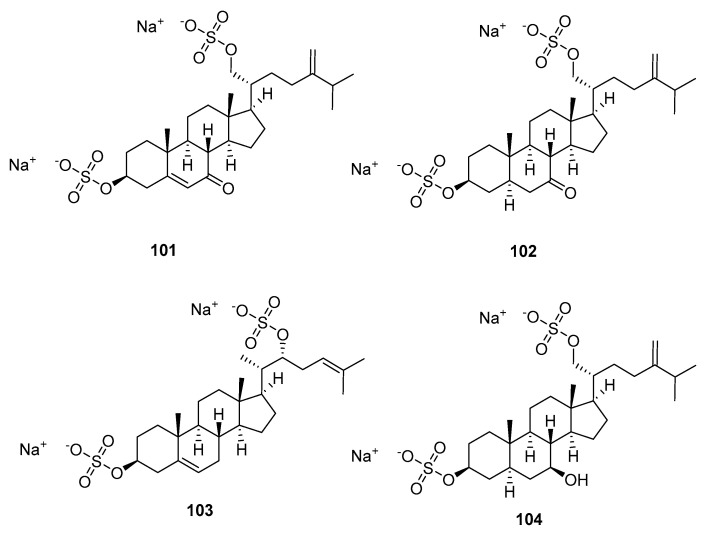
The structures of compounds **101**–**104**.

Kicha et al. isolated two new steroidal 3β,21-disulfates (compounds **101** and **102**, Figure 20) and two new steroidal 3β,22- and 3α,22-disulfates (compounds **103** and **104**) from the ethanolic extract of the Far Eastern starfish *Pteraster marsippus* [65]. The cytotoxic activities of **101** and **104**, and the mixture of **102** and **103**, were determined on the models of 2D and 3D cultures of the HEK293, SK-MEL-28, HuTu80, and ZR-75-1 cell lines. The mixture of **102** and **103** inhibited the cell viability of HEK293, SK-MEL-28, HuTu80, and ZR-75-1 by 28%, 33%, 34%, and 55%, respectively, at a concentration of 100 μM after 24 h treatment.

**Figure 21:**
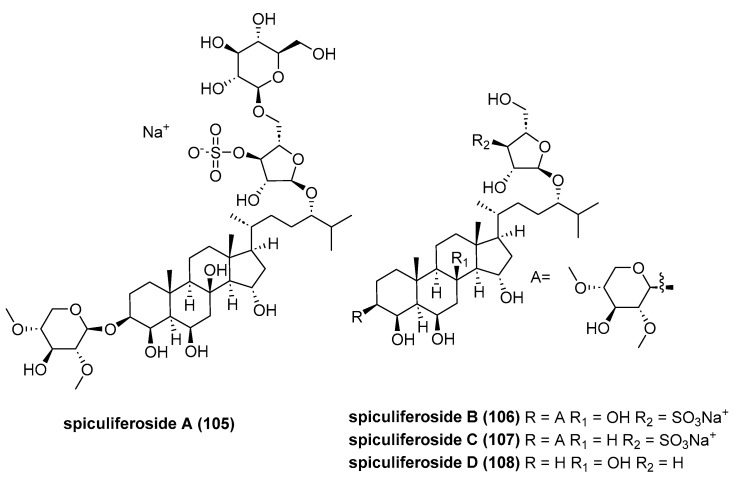
The structure of spiculiferosides A-D (**105**–**108**).

Kicha reported three new monosulfated polyhydroxysteroid glycosides, spiculiferosides A-C (**105**–**107**, Figure 21), along with a related unsulfated monoglycoside, spiculiferoside D (**108**), from an ethanolic extract of the starfish *Henricia leviuscula spiculifera*, collected in the Sea of Okhotsk [66]. Cell viability analysis revealed that compounds **105**–**107** (at concentrations up to 100 μM) exhibited negligible cytotoxicity against HEK293, SK-MEL-28, MDA-MB-231, and HCT 116 cells. However, these compounds significantly inhibited proliferation and colony formation in HCT 116 cells at non-toxic concentrations, among which spiculiferosides C showed the strongest effect. Spiculiferosides C exerted its anti-proliferative effects on HCT 116 cells by inducing dose-dependent cell cycle arrest at the G2/M phase, regulating the expression of cell cycle proteins (CDK2, CDK4, cyclin D1, and p21), and inhibiting the phosphorylation of protein kinases c-Raf, MEK1/2, and ERK1/2 in the MAPK/ERK1/2 signaling pathway.

**Figure 22:**
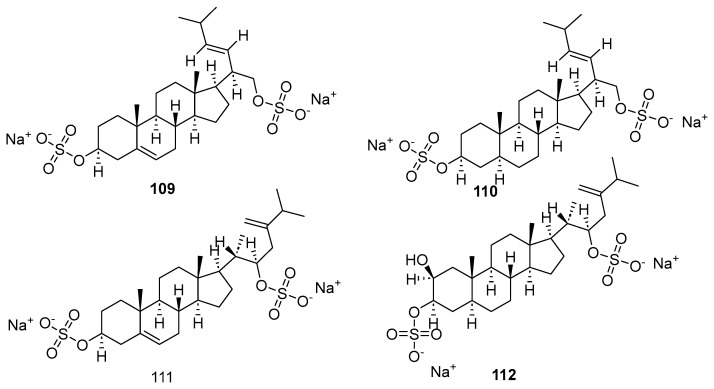
The structure of compounds **109**–**112**.

Two new steroid 3β,21-disulfates (**109**, **110**; Figure 22) and two new steroid 3β,22- and 3α,22-disulfates (**111**, **112**) were identified in the ethanolic extract of the Far Eastern slime sea star *Pteraster marsippus* [67]. Compounds **111** and **112** feature a ∆24(28)-22-sulfoxy-24-methylcholestane side chain, a structure first discovered in the polar steroids of starfish and brittle stars. The effects of compounds **109**–**112** on cell viability, colony formation, and the growth of human breast cancer cell lines T-47D, MCF-7, and MDA-MB-231 were examined. At a concentration of 50 μM, compound **112** reduced the viability of T-47D cells by 22%, while compound **111** inhibited the viability of MDA-MB-231 cells by 17%.

**Figure 23:**
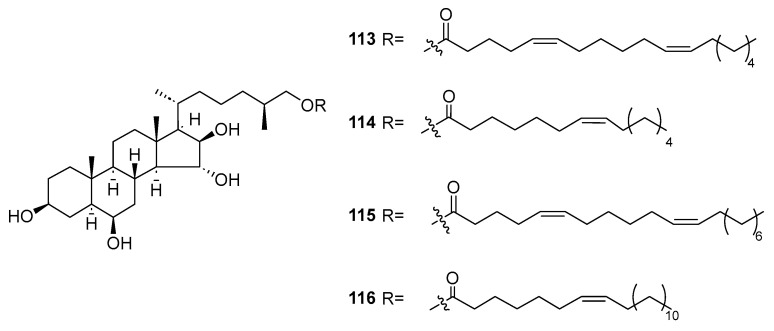
The structure of compounds **113**–**116**.

Four new conjugates, esters of polyhydroxysteroids with long-chain fatty acids (**113**–**116**, Figure 23), were isolated from the deep-water Far Eastern starfish *Ceramaster patagonicus*. The study revealed that compounds **113**–**115** elicited a 50% inhibition of growth (IC_50_) in JB6 Cl41 cells at concentrations of 81, 40, and 79 μM, respectively. In the case of MDA-MB-231 cells, the IC_50_ values for compounds **113**–**115** were determined to be 74, 33, and 73 μM, respectively. Similarly, for HCT 116 cells, the IC_50_ values for these compounds were 73, 31, and 71 μM, respectively. Notably, compound **116** displayed no toxicity against the tested cell lines, even after a three-day treatment period. Furthermore, compound **114**, at a concentration of 20 μM, significantly inhibited colony formation and migration in both MDA-MB231 and HCT 116 cells [68].

**Figure 24:**
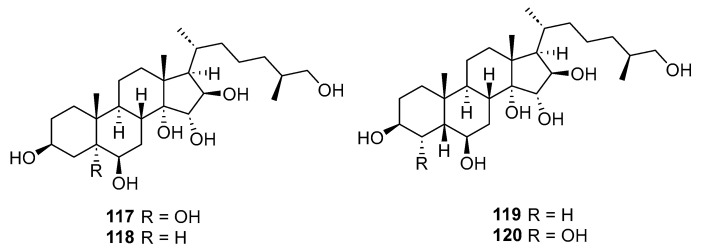
The structure of compounds **117**–**120**.

Kicha, A. A. et al. reported four new polyhydroxylated steroids **117**–**120** (Figure 24)**,** which were isolated from the methanolic extract of the starfish *Anthenoides laevigatus*, collected off the coastal waters of Vietnam [69]. Compound **117** did not show cytotoxic effects against JB6 Cl41, HT-29, or MDA-MB-231 cells; however, it decreased the colony number of HT-29 cells by 18%, and that of the MDA-MB-231 cells by 35%.

### 2.4. Substituted Aromatic Alcohols

In addition to triterpenes and steroids, echinoderms are rich in various other types of small molecules, such as substituted aromatic alcohols, terpenes, and polyketides. These molecules exhibit diverse pharmacological potential in areas such as antibacterial, anti-inflammatory, and neuroprotective activities. Certain polyketides and aromatic alcohols have demonstrated high selectivity in inhibiting specific targets, such as COX-2 and 5-LOX. Further in vivo pharmacodynamic and pharmacokinetic studies are warranted to explore their therapeutic potential.

**Figure 25:**
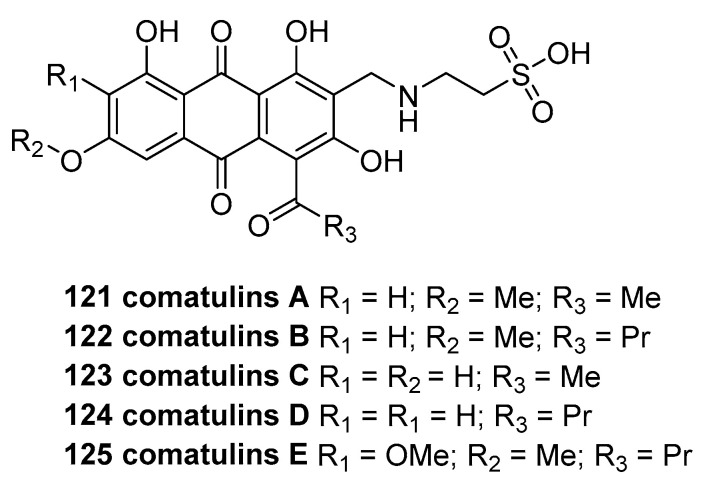
The structure of comatulins A−E (**121**–**125**).

Chemical investigations of two specimens of the Australian crinoid *Comatula rotalaria* afforded five new taurine-conjugated anthraquinones, comatulins A–E (**121**−**125**, Figure 25) [70]. Compounds A–E did not exhibit significant anti-HIV-1 replication activity or antiparasitic activity under the experimental conditions described in the study.

**Figure 26:**
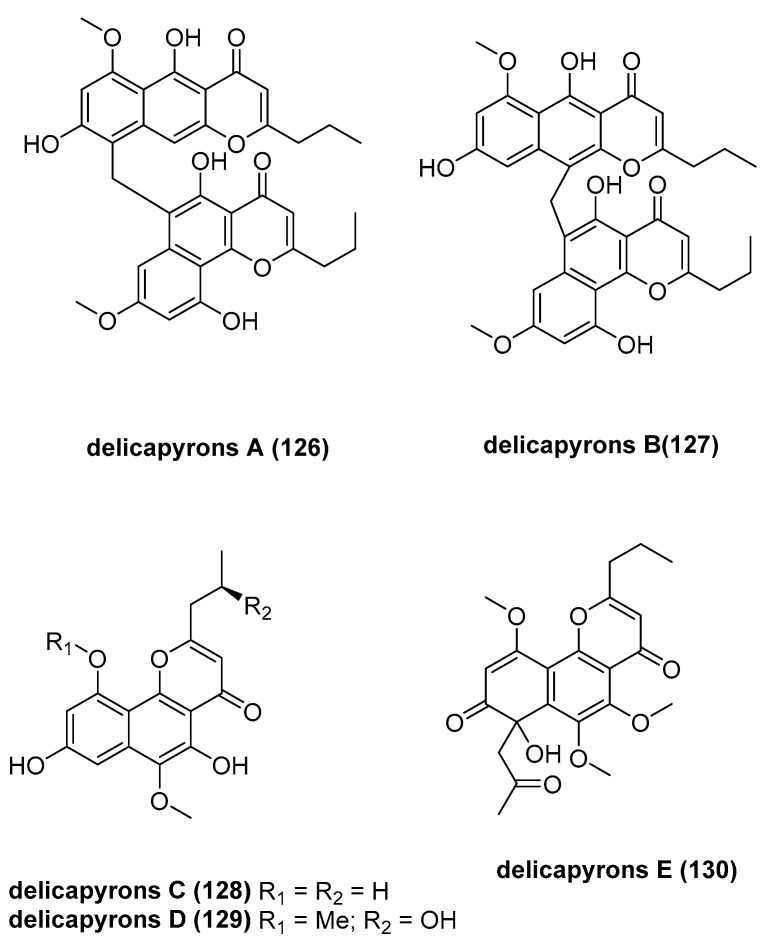
The structure of delicapyrons A-E (**126**–**130**).

Vien, L. T. reported five new naphthopyrone derivatives, delicapyrons A–E (**126**–**130**, Figure 26), and nine known compounds, which were isolated from the MeOH extract of the Vietnamese crinoid *Comanthus delicata* [71]. Among the isolated compounds, delicapyron B (**127**) exhibited significant and selective cytotoxicity against SK-Mel-2 lines, with an IC_50_ of 11.99 ± 0.69 μM.

**Figure 27:**
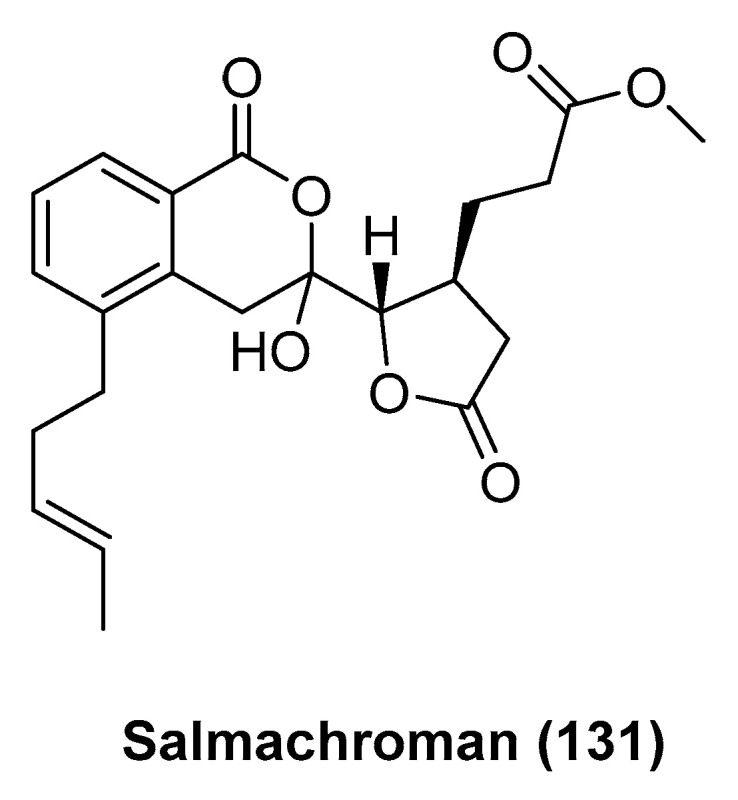
The structure of salmachroman (**131**).

Salmachroman (**131**, Figure 27), an isochroman-derived polyketide from the sea urchin *Salmacis bicolor* collected from the southeast coast of the Arabian Sea, was isolated and characterized by Francis, P. et al. [72]. Salmachroman exhibited significant dual inhibitory potential against the pro-inflammatory enzymes cyclooxygenase-2 (COX-2) and 5-lipoxygenase (5-LOX), with IC_50_ values of 1.29 μM and 1.39 μM, respectively; these were lower than those of the standard anti-inflammatory drug ibuprofen (IC_50_ for 5-LOX: 4.50 μM). Additionally, it demonstrated stronger radical scavenging activities against ABTS^+^ and DPPH free radicals, compared to the standard antioxidant α-tocopherol (IC_50_ > 1.50 μM), with IC_50_ values of 1.19 μM and 1.24 μM, respectively. Salmachroman exhibited a higher selectivity index for COX-2 inhibition compared to ibuprofen, with a selectivity index of 1.03 versus 0.43, respectively. This suggests that Salmachroman has a more pronounced preferential inhibition of the COX-2 enzyme, which is often associated with reduced gastrointestinal side effects and is a desirable characteristic in nonsteroidal anti-inflammatory drugs (NSAIDs).

**Figure 28:**
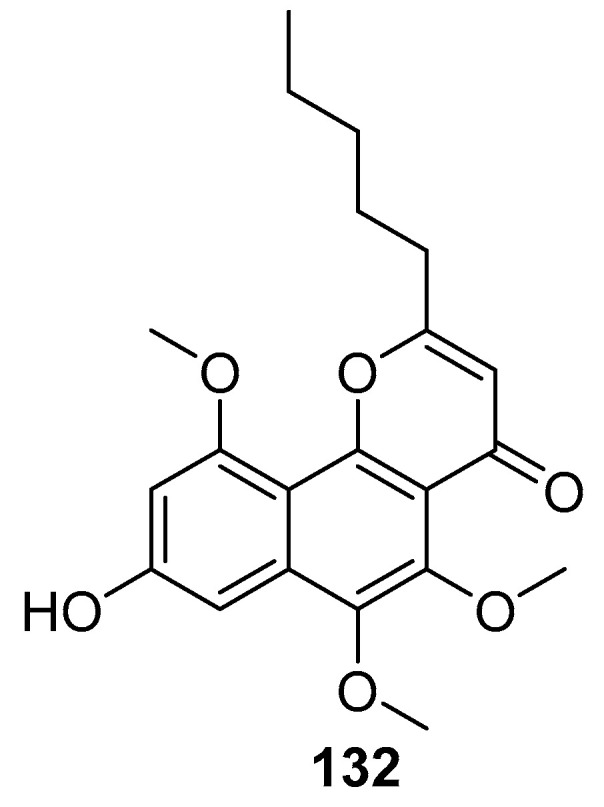
The structure of compound **132**.

Chung, H.-M. et al. reported a study on *Colobometra perspinosa* from the South China Sea that resulted in the isolation of five angular naphthopyrones, including a new metabolite. Compound **132** (Figure 28) at 10 μM exhibited a potent anti-inflammatory effect, with 83.75% iNOS inhibition in LPS-stimulated RAW264.7 macrophages [73].

**Figure 29:**
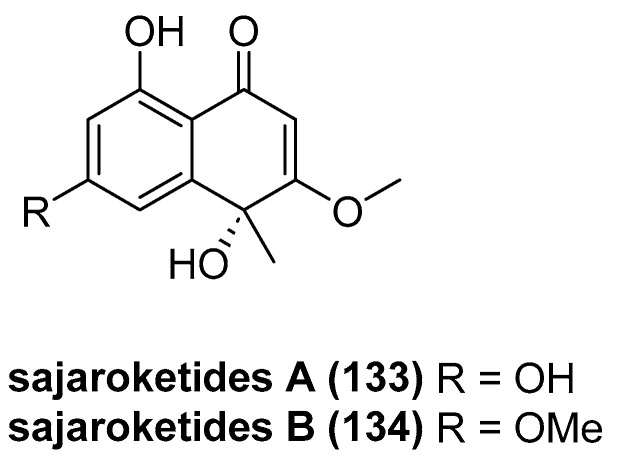
The structure of sajaroketides A (**133**) and B (**134**).

Leshchenko, E. V. et al. isolated two new polyketides from marine-derived fungal strains KMM 4718 and KMM 4747 isolated from sea urchin *Scaphechinus mirabilis* as a natural fungal complex, including sajaroketides A (**133**) and B (**134**) (Figure 29) [74]. The inhibitory effects of compounds **133** and **134** on urease enzyme activity and their impacts on the proliferation of *Staphylococcus aureus*, *Escherichia coli*, and *Candida albicans* strains were tested. Both compounds demonstrated the ability to inhibit urease activity, with IC_50_ values of greater than 100 μM for compound **133** and 98.5 μM for compound **134**, respectively. Notably, Sajaroketide A (compound **133**) at a concentration of 100 μM resulted in a 35.1% reduction in the growth of *S. aureus* and a 25.4% reduction in the growth of *E. coli*, indicating a selective inhibitory effect on these bacterial strains.

**Figure 30:**
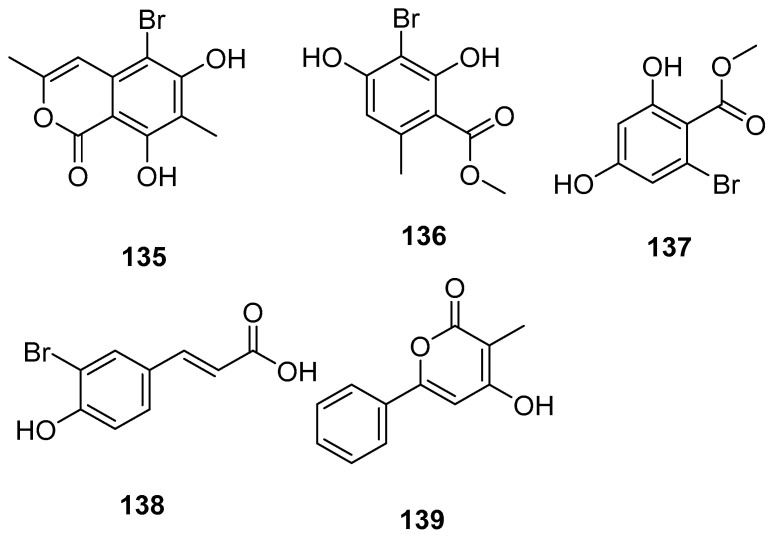
The structure of compounds **135**–**139**.

A new brominated isocoumarin, 5-bromo-6,8-dihydroxy-3,7-dimethylisocoumarin (**135**, Figure 30), along with four new natural products, methyl 3-bromo-2,4-dihydroxy-6-methylbenzoate (**136**), methyl 2-bromo4,6-dihydroxybenzoate (**137**), (E)-3-(3-bromo-4-hydroxyphenyl) acrylic acid (**138**), and 4-hydroxy-3-methyl-6-phenyl-2H-pyran-2one (**139**), were isolated from the ethyl acetate extract of the fungus *Aspergillus* sp. WXF1904 marine starfish collected from the Shishen Seamount in the Northwest Sub-basin of the South China Sea. Compound **135** was evaluated for inhibitory activity against acetylcholinesterase and pancreatic lipase. It had weak inhibitory activity against acetylcholinesterase at a concentration of 50 μg/mL, with 28.7% inhibition [75].

**Figure 31:**
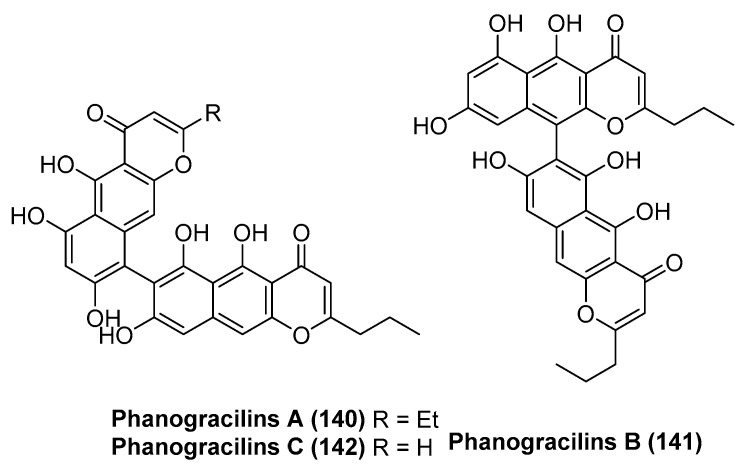
The structure of compounds **140**–**142**.

Three new bibenzochromenones named phanogracilins A–C (**140**–**142**, Figure 31) were isolated from the crinoid *Phanogenia gracilis* collected by scuba divers in the South China Sea near Ly Son Island (depth 3–9 m). Bibenzochromenones **140**–**142** were tested for their antiradical, neuroprotective, and antimicrobial activities. Compounds **140** and **142** demonstrated significant antiradical properties towards ABTS radicals, higher than the positive control Trolox, with IC_50_ values of 10.46 μM and 9.43 μM. Compounds **140** and **142** exhibited moderate neuroprotective activity, increasing the viability of rotenone-treated Neuro-2a cells at a concentration of 1 μM by 9.8% and 11.8%, respectively. Compounds **140** and **142**, at concentrations from 25 to 100 μM, dose-dependently inhibited the growth of Gram-positive bacteria *S. aureus* and yeast-like fungi *C. albicans*, and they also prevented the formation of the associated biofilms [76].

### 2.5. Others

In addition to the common terpenoids, echinoderms also contain less-common terpenoid types, as well as other compounds such as fatty acid anhydrides, macrolides, and cembranoids. These molecules have demonstrated notable activities, including antitumor and anti-inflammatory effects.

**Figure 32:**
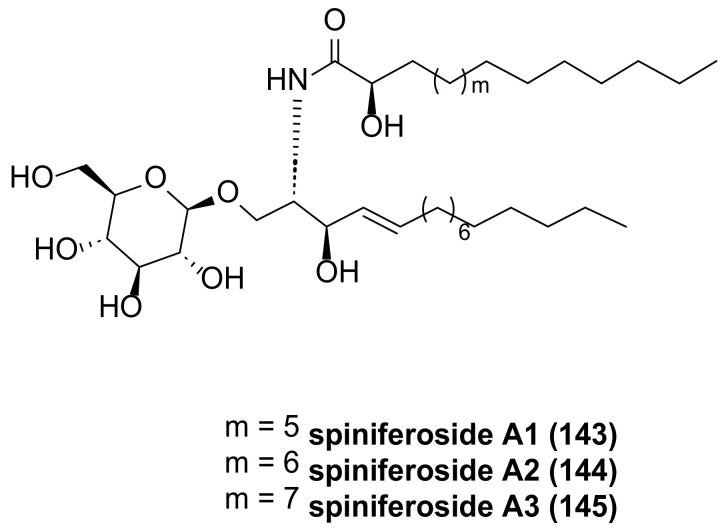
The structure of spiniferosides A-C (**143**–**145**).

Abdelhameed et al., using bioactivity-guided fractionation of a methanolic extract of the cucumber *Holothuria spinifera* from the Red Sea and LC-HRESIMS-assisted dereplication, produced the isolation of three new cerebrosides, spiniferosides A–C (**143**–**145**, Figure 32) [77]. Compounds **143**–**145** were tested for their in vitro cytotoxicities against the breast adenocarcinoma MCF-7 cell line. Compounds **143**, **144**, and **145** showed promising cytotoxic activities against MCF-7 cells, with IC_50_ values of 13.83, 8.13, and 8.27 μM, compared to that of the standard drug doxorubicin (IC_50_ = 8.64 μM).

**Figure 33:**
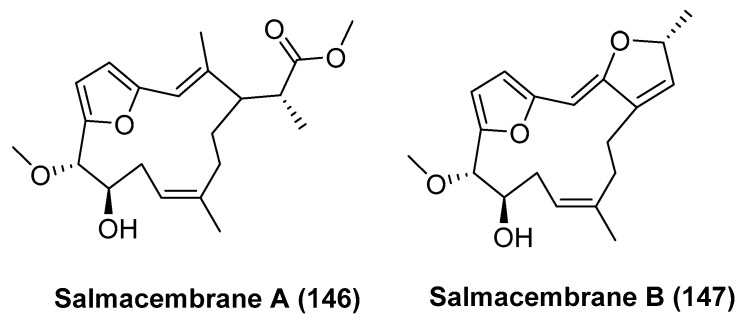
The structure of salmacembrane A (**146**) and B (**147**).

Francis, P. et al [78] isolated and identified previously unreported furanocembranoids from the sea urchin *Salmacis bicolor*. Salmacembrane A (**146**, Figure 33) displayed a significantly greater attenuation property against pro-inflammatory cyclooxygenase-2 (IC_50_ = 1.71 mM) than that exhibited by salmacembrane B (**147**, Figure 33) (IC_50_ = 1.99 mM). Salmacembrane A could potentially inhibit 5-lipoxygenase (IC_50_ = 1.87 mM), and its activity was significantly greater than that exhibited by the anti-inflammatory agent ibuprofen (IC_50_ = 4.50 mM).

**Figure 34:**
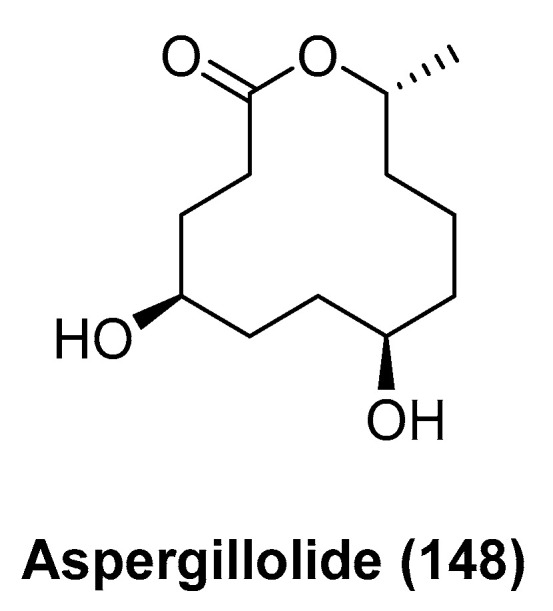
The structure of aspergillolide (**148**).

Chakraborty et al. reports the isolation of a new 12-membered macrolide named aspergillolide (**148**, Figure 34), from the fungus *Aspergillus* sp. S-3-75, which is associated with the sea cucumber *Holothuria nobilis Selenka* [79]. Compound **148** did not show activity as to the immunosuppressive effect relating to splenocyte proliferation induced by concanavalin A.

**Figure 35:**
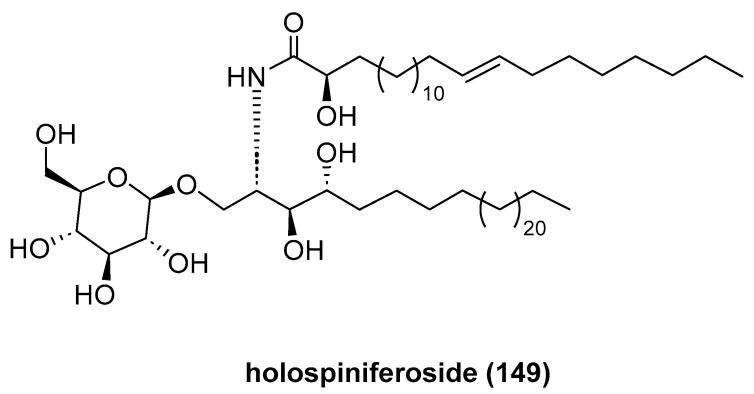
The structure of holospiniferoside (**149**).

Eltamany, E. E. et al. reported the isolation and characterization of a new compound, holospiniferoside (**149**, Figure 35), from the sea cucumber *Holothuria spinifera* [80]. Holospiniferoside exhibited a promising in vitro antiproliferative effect on the human breast cancer cell line (MCF-7), with an IC_50_ of 20.6 μM, compared to the drug cisplatin (IC_50_ = 15.3 μM).

**Figure 36:**
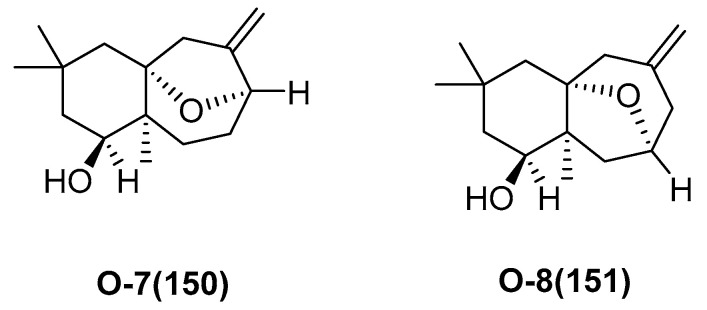
The structure of O-7 (**150**) and O-8 (**151**).

Two new sesquiterpenoids, O-7 (**150**) and O-8 (**151**) (Figure 36), were isolated from *Ophiocoma dentata*, collected from the Red Sea of Egypt [81]. Compounds showed a dose-dependent reduction in the viability of MCF-7 cells, with LC_50_ values of 103.5 and 59.5 μg/mL for compounds **150** and **151**, respectively. These values were compared to the LC50 of the chemotherapeutic agent cisplatin, which was 47.4 μg/mL. And the compounds **150** and **151** showed significant activity against *P. aeruginosa*, with absorbance units (AU) of 2.8 ± 0.05 and 2.25 ± 0.04, respectively.

**Figure 37:**
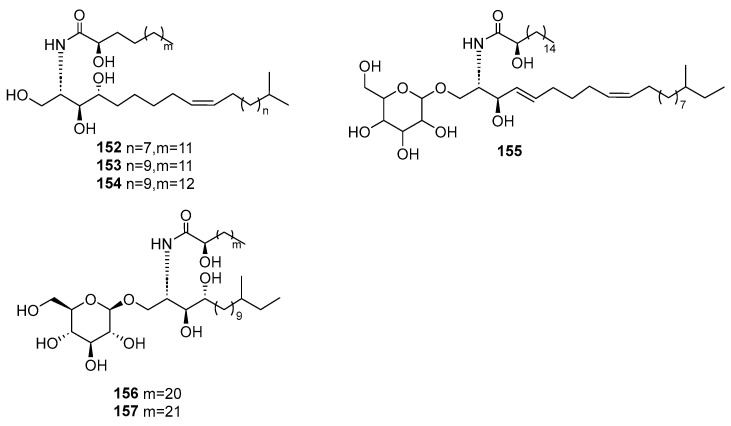
The structure of compounds **152**–**157**.

Malyarenko, T. V. et al. isolated three novel cerebrosides (**152**–**154**, Figure 37) and three new cerebrosides (**155**–**157**, Figure 37), along with three previously known cerebrosides (ophidiocerebrosides C, D [82], and CE-3-2 [83]) from the starfish *Ceramaster patagonicus*, collected at a depth of 150–300 m in the Sea of Okhotsk [84]. Compounds **152**–**157** exhibited slight to moderate cytotoxic activity against human cancer cells (HT-29, SK-MEL-28, and MDA-MB-231) and normal embryonic kidney cells HEK293. Compound **153**, at a concentration of 20 μM, inhibited colony formation of MDA-MB-231 cells by 68%.

**Figure 38:**
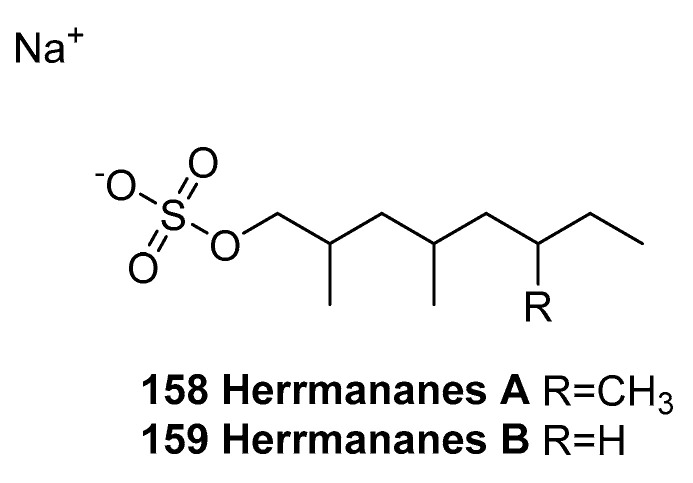
The structure of herrmananes A and B (**158** and **159**).

Vien, L. T. et al. [85] isolated two new sulfated hydrocarbons, namely, herrmananes A and B (**158** and **159**, Figure 38), from the aqueous fraction of the methanol residue of the sea cucumber *Stichopus herrmanni*. Compound **158** showed significant cytotoxicity on the SK-Mel-2 cell line, with an IC_50_ value of 23.12 ± 1.87 μM, whereas **159** exhibited a moderate effect on this cell line, with an IC_50_ value of 55.28 ± 4.17 μM, both as compared to that of ellipticine (the positive control, IC_50_ = 1.58 ± 0.20 µM). Both compounds were inactive against the four tested HepG2, KB, LNCaP, and MCF7 cell lines (IC_50_ > 100 µM).

**Figure 39:**
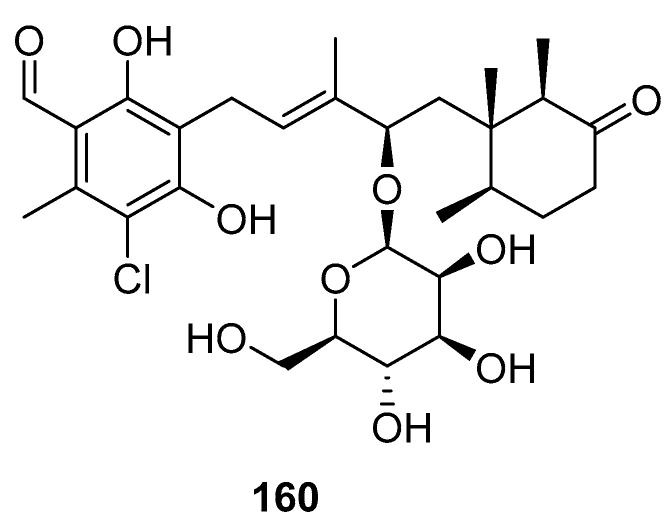
The structure of compound **160**.

A new ascochlorin glycoside (**160**, Figure 39), was successfully isolated from the fungus *Acremonium* sp. GXIMD02024, which was derived from a brittle star. The antibacterial and cytotoxicity-related activities, and the effect on α-glucosidase, of compound **160** were tested. However, upon assessment at a concentration of 40 μM, compound 160 either demonstrated negligible activity or was found to be inactive across these biological assays [86].

## 3. Conclusions and Perspective

Over the past five years, echinoderms have continued to be a rich source of diverse secondary metabolites, including terpenes and their glycosides, which play significant roles in chemical defenses against predators and pathogens. These compounds have shown pharmacological importance, with potential applications in cancer treatment, pain management, and neurological disorders. Echinoderms, including sea cucumbers and sea stars, have evolved unique chemical signatures, such as Δ7 and Δ9(11) sterols, which are not found in other animal phyla. These compounds serve as a form of chemical defense and have shown potential in various biomedical applications.

Triterpenoid saponins and stellar saponins, predominantly found in echinoderms such as sea stars and sea cucumbers, exhibit a plethora of biological activities. The broad biological activity of triterpenoid saponins is attributed to their interaction with 5,6-unsaturated sterols on the cell membrane, which induces a strong membrane disruption, leading to saponification and cell lysis [87]. Studies have also shown that the effect of the sulfate group on the membrane-promoting activity of the glycoside depends on the structure of the sugar chain and the position of the sulfate group. Hydroxyl groups attached to different positions on the aglycone side chain can significantly reduce this activity [88]. The terpenoid portion of the saponin provides the biological activity of the molecule, and highly polar sugar chains enhance the water solubility and pharmacokinetic properties [89]. The unique structures of saponins facilitate interactions with other pharmaceutical agents, enabling saponins to act as adjuvants that enhance the pharmacokinetics of drugs [90].

Current research on these compounds still faces certain limitations: most studies remain at the cellular or enzyme level, lacking animal experiments and preclinical evaluations. Triterpene and steroidal scaffolds have not undergone systematic chemical modification, making it difficult to accurately assess their active core groups. Future research should focus on the systematic exploration of echinoderm biodiversity to address global challenges in drug resistance and therapeutic innovation, thereby paving the way for new applications of marine natural products in medicine.

Looking ahead, the exploration of marine echinoderm metabolites is expected to continue the expansion of our understanding of their chemical diversity and pharmacological potential. The unique biosynthetic pathways in echinoderms, such as the production of Δ7 and Δ9(11) sterols, offer a rich source for the discovery of novel compounds with potential therapeutic applications.

The integration of advanced analytical techniques, such as 2D NMR and HR-ESI-MS, will continue to play a crucial role in the structural elucidation of these complex metabolites. Furthermore, the study of the biosynthetic pathways of these molecules in echinoderms will provide insights into their unique chemical adaptations and may lead to the development of new drugs.

The potential of echinoderm-derived metabolites in antiviral applications is also a promising area for future research, as demonstrated by the antiviral potential of sea urchin aminated spinochromes against herpes simplex virus type 1. This highlights the need for further investigation into the antiviral properties of echinoderm metabolites. In conclusion, the field of marine echinoderm secondary metabolites is rich with potential for new discoveries and applications. The continued study of these compounds will not only enhance our understanding of marine chemical ecology but also contribute to the development of new pharmaceuticals and therapeutic agents.

## 4. Methodology

### 4.1. Databases and Search Keywords

Databases searched: we primarily searched PubMed, Web of Science, and marinlit, covering publications from 2020 to the present.

Search keywords: keywords used included “echinoderms”, “bioactive compounds”, “triterpene glycosides”, “marine natural products”, and “chemical diversity”.

### 4.2. Inclusion and Exclusion Criteria

Inclusion criteria: original research articles published within the last five years that are related to the chemical components and biological activities of echinoderms, including in vivo or in vitro studies.

Exclusion criteria: review articles unrelated to echinoderms, articles lacking studies on compound structures and activities, and non-peer-reviewed papers were excluded.

### 4.3. Data Extraction and Organization

Data extracted: key compound names, sources (e.g., sea cucumbers, starfish, and sea urchins), chemical structures, activity information (e.g., cytotoxicity, antibacterial, and anticoagulant), and research methods were extracted from the included literature.

Classification: After the works from the literature were collected and organized, compounds were categorized based on structural types (e.g., alkaloids, triterpenoids and their glycosides, and steroids and their glycosides). A comprehensive discussion was conducted on their biological mechanisms and application potential.

## Data Availability

Not applicable.
